# Identification of a Non-Retinoid Opsin Ligand Through Pharmacophore-Guided Virtual Screening—A Novel Potential Rhodopsin-Stabilizing Compound

**DOI:** 10.3390/molecules30112328

**Published:** 2025-05-26

**Authors:** Miriana Di Stefano, Maria Ghilardi, Clarissa Poles, Lisa Piazza, Gian Carlo Demontis, Giulio Poli, Tiziano Tuccinardi, Marco Macchia

**Affiliations:** 1 Department of Pharmacy, University of Pisa, 56126 Pisa, Italy; miriana.distefano@farm.unipi.it (M.D.S.); maria.ghilardi4@gmail.com (M.G.); lisa.piazza@phd.unipi.it (L.P.); giancarlo.demontis@unipi.it (G.C.D.); tiziano.tuccinardi@unipi.it (T.T.); marco.macchia@unipi.it (M.M.); 2 Telethon Institute of Genetics and Medicine, 80078 Naples, Italy; c.poles@tigem.it; 3 Genomics and Experimental Medicine Program, Scuola Superiore Meridionale (SSM, School of Advanced Studies), 80078 Naples, Italy

**Keywords:** rhodopsin, molecular chaperones, consensus docking, virtual screening

## Abstract

Rhodopsin, a G-protein-coupled receptor (GPCR) comprising the protein opsin covalently linked to the chromophore 11-cis retinal, is pivotal in visual phototransduction. Mutations in the gene encoding rhodopsin (RHO) can cause opsin misfolding or reduce its stability, resulting in retinal degenerative disorders such as retinitis pigmentosa (RP). Current therapeutic strategies employing retinoid-based chaperones partially rescue the folding and trafficking of mutant rhodopsin, but are limited by inherent toxicity and instability due to photoinduced isomerization. In the present work, a pharmacophore-based virtual screening protocol combined with molecular docking and molecular dynamics simulations was employed, leading to the identification of a novel non-retinoid opsin ligand that can potentially act as a pharmacological chaperone. Biological validation confirmed that the compound **VS1** binds opsin effectively, representing a valuable starting point for structure-based optimization studies aimed at identifying new opsin stabilizers.

## 1. Introduction

The visual cycle is a fundamental process that enables photoreceptors to detect light and generate visual signals. Central to this process is rhodopsin, a G-protein-coupled receptor (GPCR) located in the outer segments of rod photoreceptor cells. Rhodopsin consists of the protein opsin covalently bound to the chromophore 11-cis-retinal (11-cis-RAL), which serves as a light-sensitive component. 11-cis-RAL is the aldehyde form of vitamin A and plays a crucial role in visual phototransduction by enabling light perception. This molecule binds specifically to opsin, forming rhodopsin in rods (or cone opsins in cones), and undergoes isomerization upon photon absorption. When light is absorbed, 11-cis-RAL undergoes isomerization to all-trans-retinal, triggering a conformational change in opsin that activates the G-protein transducin. This activation initiates a biochemical cascade that amplifies the signal and ultimately leads to the transmission of electrical impulses to the brain, resulting in visual perception [1].

The regeneration of rhodopsin is essential for sustained visual function. Following photoisomerization, all-trans-retinal must be converted back to 11-cis-RAL through a series of enzymatic reactions in the retinal pigment epithelium (RPE), allowing it to rebind to opsin and restore rhodopsin’s light-sensitive state. Any disruption in this cycle can impair vision, as seen in inherited retinal degenerative disorders such as retinitis pigmentosa (RP) [2]. RP is a genetically heterogeneous disorder affecting approximately 1 in 4000 individuals worldwide [3], making it one of the most prevalent causes of inherited blindness. Over 100 different gene [4] mutations have been identified as contributors to RP, affecting various molecular pathways essential for retinal function. Mutations in the gene encoding rhodopsin (RHO) play a significant role in disease onset and progression. These mutations often lead to structural instability and the misfolding of rhodopsin, causing protein aggregation, defective intracellular trafficking, and reduced functional stability. As a result, rod photoreceptor cells become increasingly unable to process light stimuli, ultimately undergoing apoptosis and leading to progressive visual deterioration [5].

The identification of RHO mutations associated with RP has significantly advanced our understanding of the molecular mechanisms underlying the disease, shedding light on the crucial role of rhodopsin in the visual system. Moreover, research on rhodopsin-related RP has facilitated the development of both animal models and in vitro systems, which are essential tools for investigating RP pathophysiology and evaluating potential therapeutic strategies [6]. Pharmacological chaperones have been explored as potential therapeutic agents to stabilize misfolded rhodopsin and facilitate proper protein folding and trafficking. Retinoid-based chaperones, such as 11-cis retinal and 9-cis retinal (9-cis-RAL), have demonstrated partial efficacy in restoring rhodopsin function [7]. However, their application is significantly limited due to their inherent toxicity at therapeutic concentrations and susceptibility to photoinduced isomerization, which leads to a loss of efficacy and a high risk of impairing the function of cone opsins [8]. Moreover, photoinduced isomerization can occur during protein synthesis in the endoplasmic reticulum, which can cause protein instability [9]. These findings emphasize the need for alternative non-retinoid stabilizers that can support rhodopsin folding and function while avoiding the limitations of traditional retinoid-based approaches.

To address these challenges, this study aimed to identify novel non-retinoid opsin ligands that can potentially act as pharmacological chaperones capable of stabilizing rhodopsin. A computational protocol integrating pharmacophore modeling, molecular docking, and molecular dynamics (MD) simulations was employed to predict potential opsin-binding compounds. The selected candidates were evaluated on reconstituted rhodopsin to determine their binding affinities, competition with 9-cis-RAL, and impact on rhodopsin regeneration. Experimental validation confirmed that a compound identified through virtual screening, **VS1**, binds to opsin, effectively competing with 9-cis-RAL and reducing rhodopsin regeneration in a manner comparable to β-ionone, a known opsin binder. These findings underscore the relevance of non-retinoid ligands in the development of novel therapeutic strategies for rhodopsin-associated retinal disorders.

## 2. Results and Discussion

**Figure 1:**
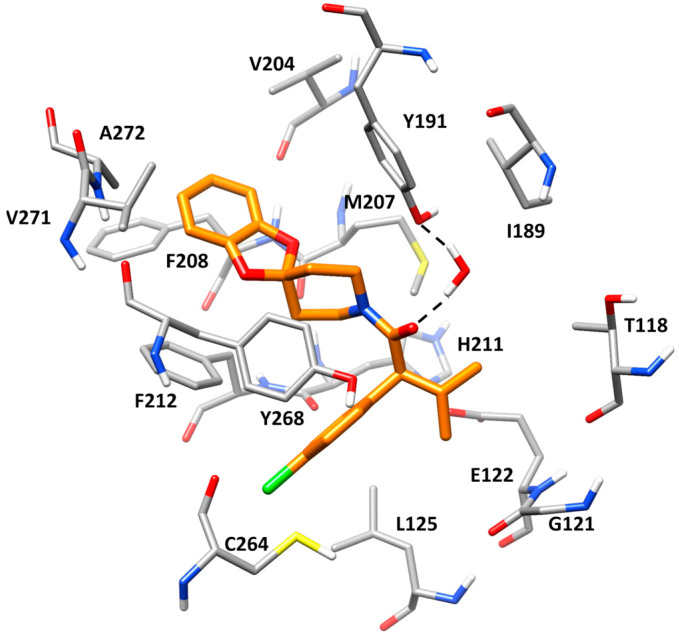
Stabilizing ligand RS01 in the retinal binding pocket of bovine rhodopsin (PDB code: 6FK6). The protein residues forming the binding site are shown as gray sticks, while H-bonds are shown as black-dashed lines.

The X-ray structure of bovine rhodopsin in complex with the stabilizing ligand RS01 (PDB code 6FK6) [10] was used as a reference in the present study. The crystallographic complex reveals that the co-crystallized ligand forms key hydrogen bonds and hydrophobic interactions within the retinal binding site. The 4-chlorophenyl moiety is embedded in a hydrophobic pocket, establishing extensive van der Waals interactions with L125, C264, and Y268 (Figure 1). The carbonyl group of the ligand is positioned within the binding pocket, forming a hydrogen bond with a structural water molecule. This water, in turn, mediates a hydrogen bond network with Y191, which is supposed to significantly contribute to the stabilization and binding affinity of the ligand within the receptor. Additionally, the isopropyl group resides in a hydrophobic region of the binding pocket, where it engages in lipophilic interactions with T118, E122, and L125, reinforcing ligand accommodation within the receptor. The phenyl ring of the benzodioxole core is positioned on the opposite side of the binding pocket, where it engages in strong lipophilic interactions with A272, F208, F212, V204, Y268, and M207.

**Figure 2:**
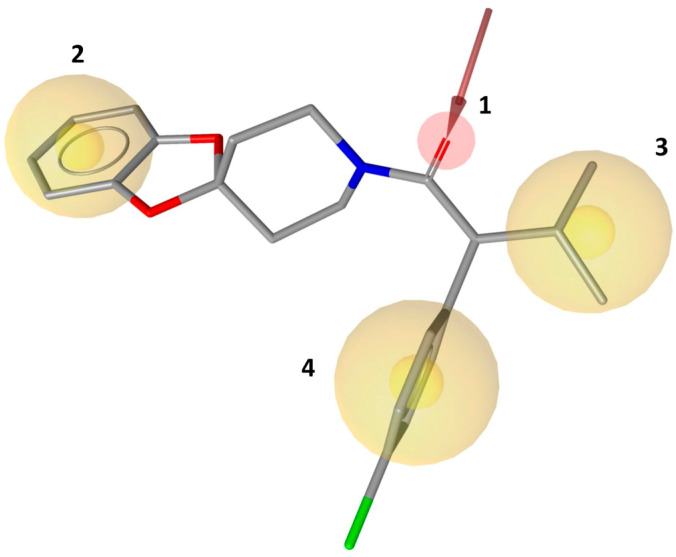
Receptor-based pharmacophore model with mandatory features 1–4 superimposed with the stabilizing ligand RS01 in the rhodopsin X-ray complex.

Based on the main ligand–protein interactions observed in the reference crystallographic complex, a receptor-based pharmacophore model was developed using Ligand Scout software [11]. The generated model comprised four pharmacophore features (Figure 2): three hydrophobic features, representing the key lipophilic interactions identified at the binding site, and an H-bond acceptor feature, representing the interaction with the structural water molecule. Moreover, the excluded volume spheres that resemble regions of space in the proximity of the pharmacophore model occupied by the protein were also taken into consideration for the pharmacophore model development. The receptor-based pharmacophore model was subsequently employed to screen a database of over 1.3 million commercial compounds, aiming to identify small molecules capable of replicating the key interactions observed at the rhodopsin binding site with the reference ligand. A total of 108,857 ligands were identified through virtual screening and subsequently subjected to consensus docking analyses. Consensus docking is a well-validated in silico protocol based on a combination of multiple docking methods to predict the potential binding mode of small molecules. For each compound subjected to this approach, multiple docking results are thus obtained and then clustered based on their reciprocal RMSD to identify the most populated binding pose (the consensus pose). Our previous studies proved that this approach is able to reproduce experimental ligand binding poses better than single docking methods, and that compounds achieving a high consensus level (i.e., compounds for which a high number of docking methods predict the same binding mode in the target receptor) are more likely to actually bind the receptor compared to others for which a low consensus level was achieved (i.e., for which only a low number of docking methods predicted the same binding mode) [12]. This strategy was applied to the 108,857 ligands identified through pharmacophore screening. However, to optimize computational efficiency, a hierarchical consensus docking approach was employed. Initially, compounds were docked using a set of six rapid docking methods, and a first pose consensus analysis was then performed to check for similar binding modes (see Materials and Methods for details). Only molecules reaching a minimum consensus level of 4 were selected for further analyses, thus retaining only 15,251 compounds. These compounds were indeed ligands for which the majority (at least four out of six) of the docking methods predicted the same binding pose.

The selected molecules were then docked into the protein binding site using five additional docking procedures, thus obtaining a total of eleven different binding dispositions for each compound, considering also the six binding poses generated in the previous consensus docking stage. The pose consensus analysis was then repeated in order to identify compounds achieving a high consensus level (i.e., compounds statistically more likely to actually bind to the receptor). Since 21 compounds achieved the maximum consensus level, because all eleven docking procedures predicted the same binding mode, these ligands were further considered. Although only a very small number of compounds were selected using this approach considering the total number of ligands evaluated, these results are in line with our previous studies where the consensus approach was used in virtual screening, which demonstrated that only a very small percentage of compounds are able to reach high consensus levels [13], and sometimes no compound even achieves full consensus (i.e., the docking methods do not predict the same binding mode for any ligand) [14].

**Figure 3:**
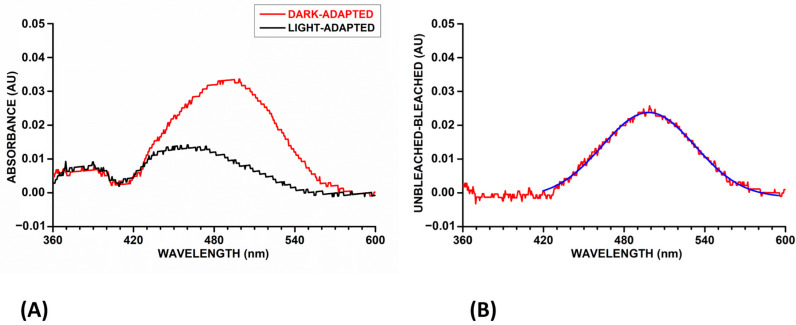
(**A**) Absorption spectra of rhodopsin recorded in the 360–600 nm range. The spectrum of dark-adapted rhodopsin is shown in red, while the light-adapted spectrum is displayed in black. (**B**) The difference spectra (red), obtained by subtracting the light-adapted spectrum from the dark-adapted spectrum were fitted with a Gaussian function (blue) to quantify rhodopsin regeneration, calculated as the area under the curve (AUC).

The selected compounds were then subjected to molecular dynamics (MD) simulations to evaluate the stability of their predicted binding within opsin. The 21 predicted ligand–protein complexes were thus subjected to a 100 ns MD protocol; the results were then analyzed in terms of the RMSD of ligand disposition during the simulation, with respect to the initial coordinates. Compounds that exhibited an average RMSD value below 2.0 Å, while maintaining the hydrogen bond with Y191 through the structural water molecule, were prioritized. Specifically, a score of 2, 1, or 0 was attributed to the compounds that respected both, only one, or none of the two filtering criteria (RMSD and H-bond), respectively. Among the 21 analyzed compounds, 7 ligands achieved a score of 2 and successfully passed the MD filter (Supplementary Table S1). These selected molecules were then processed using MolBook UNIPI version 1.6 [15] to screen for potential pan-assay interference compound (PAINS) alerts. Since no structural alerts were identified, the compounds were purchased and subsequently subjected to biological assays to evaluate their binding affinity and potential ability to stabilize the protein. Specifically, a protocol for rhodopsin regeneration was developed and optimized using UV/VIS spectroscopy. To assess the assay’s reliability, β-ionone, a well-characterized opsin binder, was used as a reference compound. Rhodopsin regeneration depends on the binding of 11-cis retinal to opsin, a process that can be quantitatively monitored through its characteristic absorption spectrum. This study employed 9-cis-RAL, a commercially available structural analog produced by retinal pigment epithelial cells, to regenerate rhodopsin [16]. Rhodopsin was regenerated from pig retinas by incubating them with 9-cis-RAL (20 µM) in darkness. After this dark incubation phase, the samples were exposed to room light to induce rhodopsin photoisomerization. Absorption spectra were recorded in the 360–600 nm range before and after light exposure, allowing the evaluation of rhodopsin regeneration by subtracting the light-adapted spectrum from the dark-adapted spectrum (Figure 3A). The resulting difference spectrum was fitted with a Gaussian function (blue curve in Figure 3B), enabling the quantification of rhodopsin regeneration through the area under the curve (AUC).

**Figure 4:**
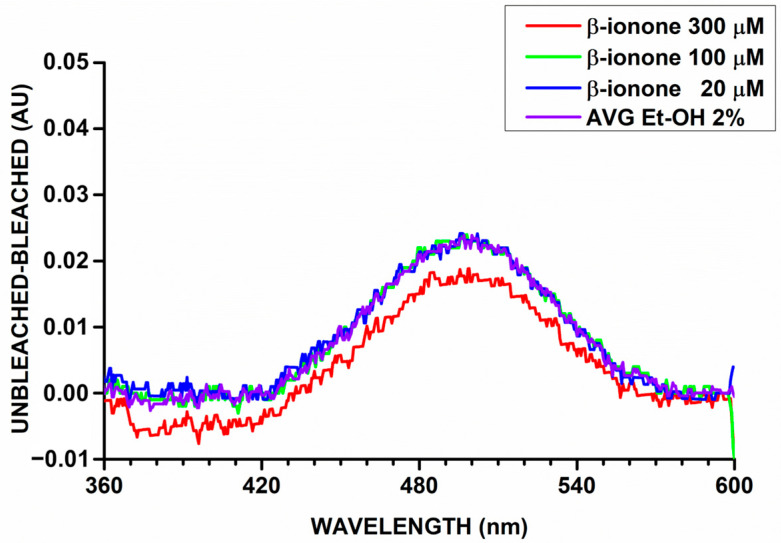
Effect of β-ionone on rhodopsin regeneration.

The AUC served as a metric to quantify rhodopsin levels and assess the impact of β-ionone, a known competitor of retinal binding. To validate the protocol and ensure its reliability, we tested three different concentrations of β-ionone to evaluate its effect on rhodopsin regeneration. At 300 µM β-ionone, a significant reduction in AUC was observed (Figure 4), confirming that β-ionone effectively competes with 9-cis-RAL for the opsin binding site, thereby decreasing rhodopsin formation. At lower concentrations, the effect was comparable to the control (2% ethanol as the solvent). These results demonstrate that the assay is highly sensitive and capable of detecting ligand competition, validating its suitability for studying rhodopsin regeneration.

The seven compounds selected based on the virtual screening study (**VS1-7**) were subjected to the same assay protocol to evaluate their ability to bind opsin. Unfortunately, due to solubility limitations in the assay solvent, only three out of seven compounds (**VS1**, **VS3**, and **VS5**) could actually be tested under the validated assay conditions. In particular, we tested each compound at the highest achievable concentration in ethanol, ensuring a minimum concentration of 50 µM (not achievable with the other four compounds). Among them, **VS1** was the only compound that exhibited a significant inhibitory effect on the binding of 9-cis-RAL to rhodopsin (Supplementary Figure S1). The observed effect was comparable to that of β-ionone, even though **VS1** was effective at a lower concentration (Table 1). In contrast, **VS3** and **VS5** also reduced rhodopsin formation, as indicated by a slight decrease in AUC. However, their effect was less pronounced than that of **VS1**, suggesting a weaker competition with 9-cis-RAL for binding to opsin.

**Figure 5:**
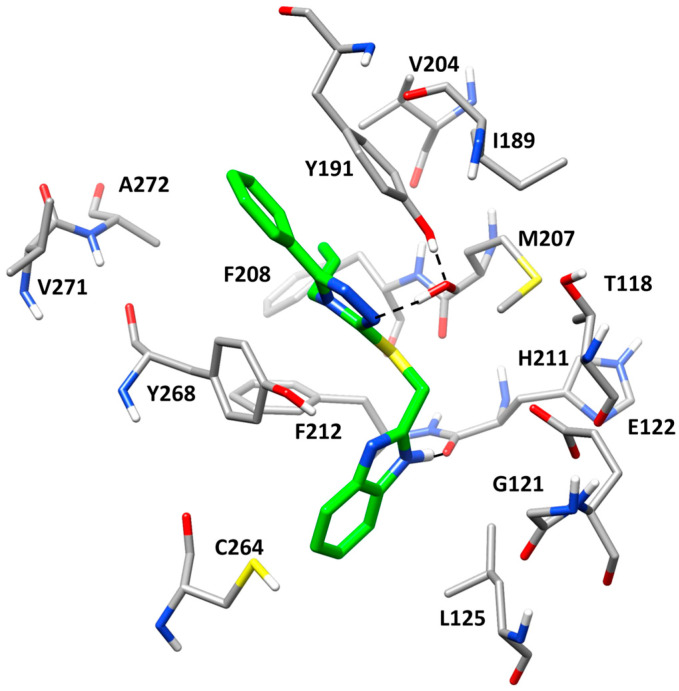
Predicted binding mode of **VS1** into opsin. Ligand–protein H-bonds are shown as black-dashed lines.

These findings demonstrate that the assay effectively measures rhodopsin regeneration and can detect ligand competition. The ability of **VS1** to inhibit rhodopsin regeneration similarly to β-ionone suggests that it competes with retinal for binding to opsin, making it a promising candidate for further investigation as a potential molecular chaperone and rhodopsin stabilizer. Figure 5 shows the predicted binding mode of compound **VS1** in complex with opsin. The ligand establishes key interactions within the binding pocket, contributing to its stabilizing effect. One of the nitrogen atoms of the triazole ring engages in an H-bond network with a structural water molecule, which in turn mediates a stable hydrogen bond with Y191, effectively anchoring the ligand within the binding site. Additionally, the benzimidazole moiety forms a direct and persistent H-bond with H122, further stabilizing the ligand–receptor complex. The phenyl group engages in a face-to-face π-π stacking interaction with Y191 and inserts into the same hydrophobic pocket occupied by the reference ligand, interacting with V204, F208, F212, Y268, V271, and A272. On the opposite side, the benzimidazole moiety of **VS1** extends in the other hydrophobic cavity formed by G121, L125, and C264. Binding free energy evaluations were also performed using the molecular mechanics/Poisson–Boltzmann surface area (MM-PBSA) approach [17], which estimated a binding energy of −13.7 kcal/mol for the **VS1**–opsin complex. Interestingly, binding free energy values of −11.2 and 12.3 kcal/mol were estimated for **VS3** and **VS5**, respectively, in complex with opsin. Although only slight differences in the binding energy values of the three ligands were predicted, these results support the experimental evidence highlighting **VS1** as the strongest opsin binder among the three tested compounds.

## 3. Materials and Methods

### 3.1. Pharmacophore Model Generation

The pharmacophore model was created using LigandScout 4.2 [11]. The pharmacophore hypothesis was built from the X-ray structure of rhodopsin in complex with (2~{S})-2-(4-chlorophenyl)-3-methyl-1-spiro[1,3-benzodioxole-2,4′-piperidine]-1′-yl-butan-1-one inhibitor, referred to as RS01 (PDB code: 6FK6) [10]. An exhaustive pharmacophore model including all possible features recognized by the program was constructed and, subsequently, only the desired features were taken into consideration in the final pharmacophore model, with a total of three hydrophobic features and one H-bond acceptor feature. Moreover, the excluded volume spheres that represent regions of space in the proximity of the pharmacophore model occupied by the enzyme were also taken into consideration for the pharmacophore model development.

### 3.2. Database Generation and Pharmacophore Screening

Approximately 1.3 million compounds from the Vitas-M commercial database were utilized as the screening database. The iCon [18] software, integrated within LigandScout, was employed for ligand conformational sampling and to configure the screening database. The previously developed pharmacophore model, containing excluded volume spheres, was employed to filter the screening database and identify compounds possessing the desired properties. Only the compounds that precisely matched all 4 features of the model were retrieved and selected for subsequent investigations.

### 3.3. Hierarchical Consensus Docking Analysis

All docking calculations were performed using the X-ray structure of rhodopsin (PDB code: 6FK6) already employed for pharmacophore modeling. To enhance computational efficiency, a hierarchical consensus docking approach was implemented. Initially, a set of six fast docking procedures was employed—Autodock Vina 1.1, Fred 3.0, Plants 1.2, rDOCK, and Gold 5.1 (using ChemScore and ChemPLP fitness functions)—following previously validated protocols [12]. By applying the six docking methods, six different binding dispositions (best-scored docking pose) resulted from the docking of each ligand into the protein binding site. The RMSD of each docking pose against the remaining five was evaluated using the rms_analysis software of the Gold suite. On this basis, a 6 × 6 matrix was generated reporting the RMSD results for each ligand. By using an in-house program [12], these results were clustered, so that among the five results, all similar docking poses were clustered together. As a clustering algorithm, we used the complete-linkage method, which is an agglomerative type of hierarchical clustering. We selected an RMSD clustering threshold of 2.0 Å; therefore, the as-obtained clusters contained the group of poses that were less than 2.0 Å away from all others poses belonging to the same cluster. At the end of this first pose consensus analysis, only ligands showing a minimum consensus level of four (i.e., those for which at least four out of the six docking programs predicted the same binding pose) were taken into account.

After this preliminary analysis, the process continued with the integration of additional docking procedures, including Gold 5.1 (using GoldScore and Astex Statistical Potential fitness functions), DOCK 6.7, Autodock 4.2.3, and GlamDock 1.0, applied as previously described [12]. The compounds selected through the initial docking stage were docked using the additional five docking procedures, thus obtaining a total of eleven different binding dispositions for each ligand. The pose consensus analysis was then repeated, generating an 11 × 11 matrix of docking poses for each ligand and clustering the poses using the same RMSD threshold (2.0 Å). At the end of the second pose consensus analysis, which concluded the hierarchical approach, only compounds with the maximum consensus level were further considered.

### 3.4. Molecular Dynamics Simulations

The software AMBER, version 20 [19], was used for the MD studies. The ligands were parameterized using General Amber force field (GAFF), with partial charges calculated based on the AM1-BCC method, as implemented in the Antechamber suite. All ligand–protein complexes were solvated with a 10.0 Å water cap and enclosed in a rectangular parallelepiped water-box. The TIP3P explicit solvent model was used for this purpose, and either Na⁺ or Cl⁻ ions were added as appropriate to ensure the systems’ neutrality. Prior to the MD simulations, two stages of energy minimization were performed. In the first one, a harmonic potential of 10 kcal/(mol·Å²) was applied to the solute; this wat only the position of the water molecules was minimized. A total of 5000 steps of the steepest descent algorithm, followed by the conjugate gradient, were applied in this stage, until a convergence of 0.05 kcal/(mol·Å²). The second step was performed applying the same steepest descent and conjugate gradient steps, but only the protein α-carbons were subjected to a position restraint of 10 kcal/(mol·Å²), thus energy minimizing the whole systems. Periodic boundary conditions and Particle Mesh Ewald (PME) electrostatics were used in all simulations. The minimized complexes were employed as the starting conformations for the MD simulations, which were performed using a time step of 2.0 fs, a cut-off of 10.0 Å for the non-bonded interactions, and keeping all bonds involving hydrogen atoms rigid using SHAKE algorithm. An initial 1.0 ns MD simulation performed with constant volume was used to raise the temperature of the systems from 0 to 300 K. A 4 ns constant-pressure MD simulation, performed using the Langevin thermostat to maintain the temperature constant, was then applied to equilibrate the systems. Finally, 95 ns of constant pressure periodic boundary MD was performed at 300 K using the Langevin thermostat. Therefore, a total of 100 ns of MD simulation was performed for each system. All the α-carbons of the protein were subjected to a position restraint of 10 kcal/(mol·Å²) during the whole MD simulation. All the obtained MD trajectories were analyzed using the cpptraj program implemented in AMBER 20.

### 3.5. Biological Evaluation of Identified Compounds

Compounds **VS1**–**VS7** were purchased from Vitas-M Corporation and tested on pig retinas. Pig eyes were obtained from a local slaughterhouse from animals being sacrificed for commercial purposes, stored on ice, and processed within a few hours post-mortem. All chemicals and reagents were obtained from Sigma-Aldrich. Retinas isolated under dark conditions were stored at −20 °C for later use. On the day of the assay, two pig retinas were homogenized in 2 mL of phosphate-buffered saline (PBS) with Ca^2+^ and Mg^2+^ (Thermo Fisher, Waltham, MA, USA) using a 2.5 mL glass/glass potter. Light-adapted homogenate aliquots were incubated with test compounds at 28 °C for 1 h, and then incubated in darkness with 9-cis-RAL at a final concentration of 20 µM to regenerate rhodopsin. β-ionone at 300 µM was used as a reference control. The test compounds were dissolved at their maximum solubility in ethanol (final solvent concentration 2% EtOH). At the end of incubation, the detergent n-dodecyl β-D-Maltoside was added to the 0.1% *w*/*v* final concentration. After detergent incubation for 40 min in darkness at 28 °C, samples were centrifuged at 3000 rpm (1500× *g*) for 10 min at 4 °C. Rhodopsin regeneration was quantified using UV/VIS spectroscopy, recording the difference absorption spectra in the 360–600 nm range to assess the binding of 9-cis-RAL to opsin in the presence and absence of competing compounds. Following the initial spectroscopic analysis, samples were then exposed to light for 1 h to induce the photobleaching of rhodopsin, followed by a second UV/VIS measurement. The difference between the dark- and light-adapted spectra was fitted with a Gaussian function using Microcal Origin Pro 8.5.1 to compute the area under the curve (AUC), which was used to quantify rhodopsin regeneration and assess the test compounds’ ability to compete with 9-cis-RAL for opsin binding.

### 3.6. Binding Energy Evaluations

The results of the MD simulations obtained for the corresponding ligand–protein complexes were used as input for the binding free energy evaluations, which were performed based on a previously validated protocol [20]. In particular, a total of 100 snapshots were extracted, at time intervals of 1 ns, from the MD trajectories and used for the evaluations. Van der Waals electrostatic and internal interactions were calculated with the SANDER module of AMBER 20, and the MOLSURF program was employed to estimate the nonpolar energies. Polar energies were calculated using the Poisson–Boltzmann method with the MM-PBSA module of AMBER 20.

## 4. Conclusions

A receptor-based virtual screening protocol was developed to identify novel non-retinoid opsin ligands that could potentially act as molecular chaperones and rhodopsin stabilizers. The workflow combined pharmacophore modeling, molecular docking, and molecular dynamics simulations to prioritize candidates mimicking key interactions within the rhodopsin binding pocket. Using the X-ray structure of rhodopsin complexed with a stabilizing ligand, a receptor-based pharmacophore model guided the selection of seven compounds. Biological evaluations using UV/VIS spectroscopy assessed rhodopsin regeneration. Among them, **VS1** significantly inhibited 9-cis-RAL binding to opsin, indicating its ability to interact with the protein and prevent rhodopsin formation. This demonstrates the ability of **VS1** to bind opsin at the level of the protein binding site, which could endow the ligand with potential rhodopsin-stabilizing activity and confirms its promise as a lead compound. **VS1** could serve as a starting point for structure-based optimization studies aimed at developing new non-retinoid opsin stabilizers. Future in silico and experimental studies will be aimed at further evaluating and characterizing the opsin binding profile of **VS1** and its potential activity as a molecular chaperone and rhodopsin-stabilizing compound.

## Figures and Tables

**Table 1:** AUC values observed for rhodopsin regeneration in presence of 9-cis-RAL and **VS1** (200 µM), **VS3** (100 µM), **VS5** (80 µM), β-ionone (300 µM). The AUC value observed in the presence of 9-cis-RAL only (control) is also reported.

#	Structure	AUC ± SD
**VS1**	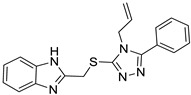	0.78 ± 0.15
**VS3**	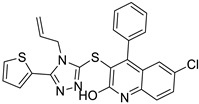	0.92 ± 0.17
**VS5**	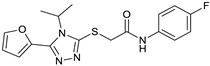	0.90 ± 0.10
**β-ionone**	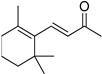	0.73 ± 0.27
**9-cis-RAL**	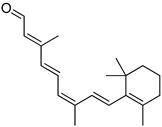	1 ± 0.07

## Data Availability

Data are contained within the article.

## Supplementary Materials

The following supporting information can be downloaded at: https://www.mdpi.com/article/10.3390/molecules30112328/s1, Table S1: Scores obtained by the 21 compounds studied with MD simulations; Figure S1: Effect of **VS1** on rhodopsin regeneration.

## Abbreviations

The following abbreviations are used in this manuscript:

GPCR	G-protein-coupled receptor
RHO	rhodopsin
RP	retinitis pigmentosa
11-cis-RAL	11-cis-retinal
9-cis-RAL	9-cis-retinal
RPE	retinal pigment epithelium
MD	molecular dynamics
MM-PBSA	molecular mechanics/Poisson–Boltzmann surface area
AUC	area under the curve
GAFF	general Amber force field
PME	Particle Mesh Ewald
PBS	phosphate-buffered saline
