# Supplementary material for: Identification of a Non-Retinoid Opsin Ligand Through Pharmacophore-Guided Virtual Screening—A Novel Potential Rhodopsin-Stabilizing Compound
Source: Molecules. 2025 May 26;30(11):2328. doi: 10.3390/molecules30112328 (PMC12155680; doi:10.3390/molecules30112328)
Supplement: Supplementary file 1 [file molecules-30-02328-s001.zip › molecules-3619989-supplementary.pdf]

Supporting Information

Table S1. MD scores obtained by the 21 compounds evaluated through molecular dynamics (MD) simulations, based on average ligand RMSD during the MD (RMSD) and stability of water mediated H-bond with Y191 (H-bond). Selected compounds (MD score = 2) are identified as **VS1-7**.

CPD #	RMSD	H-bond	MD Score
**VS1**	1	1	2
**VS2**	1	1	2
**VS3**	1	1	2
**VS4**	1	1	2
**VS5**	1	1	2
**VS6**	1	1	2
**VS7**	1	1	2
**8**	1	0	1
**9**	1	0	1
**10**	1	0	1
**11**	1	0	1
**12**	1	0	1
**13**	1	0	1
**14**	1	0	1
**15**	1	0	1
**16**	1	0	1
**17**	1	0	1
**18**	0	0	0
**19**	0	0	0
**20**	0	0	0
**21**	0	0	0

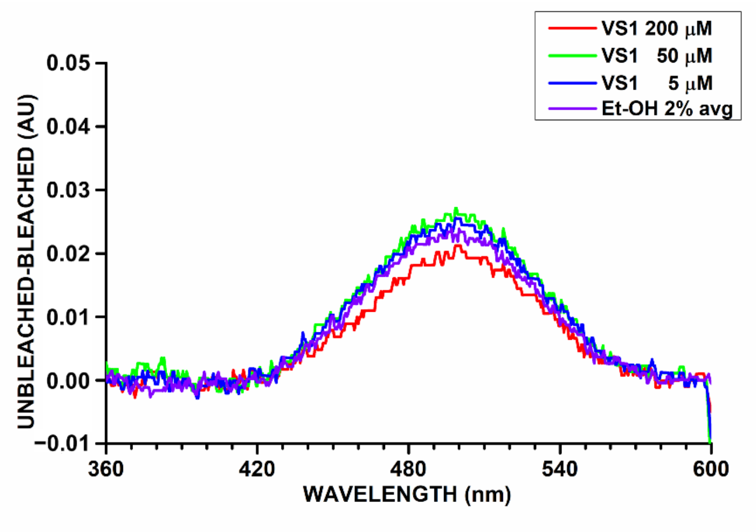

Figure S1. Effect of VS1 on rhodopsin regeneration
